# Himpathy and status: Attitudes to social hierarchy predict reactions to sexual harassment

**DOI:** 10.1371/journal.pone.0292953

**Published:** 2023-12-06

**Authors:** Morgan Weaving, Nick Haslam, Cordelia Fine

**Affiliations:** 1 School of Historical & Philosophical Studies, The University of Melbourne, Victoria, Australia; 2 Melbourne School of Psychological Sciences, The University of Melbourne, Victoria, Australia; University of Padova, ITALY

## Abstract

In three studies, we tested whether hierarchical preferences could explain differences in punishment recommendations for sexual harassment. Building on research that suggests punishment is used to regulate social hierarchies, we argue that individuals who are motivated to maintain existing hierarchies will treat male perpetrators of sexual harassment with greater leniency, especially when judging perpetrators of high social status. Conversely, we predict that egalitarians—who are motivated to reduce group-based hierarchies—will judge male perpetrators more harshly, especially those of high social status. Given competing theories in the existing literature, we make no predictions about how perpetrator status will affect punishment recommendations overall. Supporting our hypotheses, we found that individuals high on gender system justification and social dominance orientation recommended more lenient punishments to perpetrators. Moreover, an integrative data analysis uncovered an interaction between social dominance orientation and perpetrator status. This interaction was primarily driven by egalitarians, who provided more lenient punishment recommendations to low status perpetrators when compared to high status perpetrators. Contrary to our predictions, we did not find strong evidence that individuals high on social dominance orientation provided harsher judgements to low status perpetrators. Nor did we find strong evidence for a main effect of perpetrator status on punishment recommendations. Taken together, these findings suggest that people punish sexual harassment to bolster or attenuate power structures. This is particularly true of egalitarians, whose emphasis on social equality leads them to judge high status perpetrators of sexual harassment with particular severity.

## Introduction

As #MeToo unfurled, it brought a reckoning to high-status men, resulting in hundreds of high-profile resignations, litigations, and ‘cancellations’ [1]. Many believed these punishments were fair and just―symbols of a public shift in power that allowed victims to hold perpetrators accountable [2]. Others saw the punishments as excessively harsh over-corrections for historical power imbalances [3].

What can explain these differing views on punishing sexual harassment? The current studies investigated whether this variation can be explained, in part, by differing hierarchical preferences. Research suggests that individuals use punishment to regulate social hierarchies—harshly punishing those perceived to deserve a status downgrade, whilst finding greater compassion for individuals whose status is seen as important to protect [4, 5]. Consequently, those motivated to maintain patriarchal gender norms may treat perpetrators of sexual harassment with greater leniency, to protect their social status and reinforce male dominance. In contrast, those who aim to subvert hierarchical gender relations may be motivated to treat perpetrators with greater severity.

Furthermore, if punitive responses to sexual harassment are used to regulate hierarchies, the effects of hierarchical preferences may vary depending on the perpetrator’s status. Specifically, those who aim to maintain hierarchical structures should be particularly likely to treat high-status perpetrators of sexual harassment with leniency. However, the reverse should be true for those with egalitarian goals, who may treat high-status perpetrators more severely, to redress perceived inequalities and ensure high-status offenders face meaningfully negative consequences. The present investigation examined the above hypotheses over three studies, to better understand divergent responses to sexual harassment and their function.

## Punishment as hierarchy regulation

The retributive justice literature proposes that punishment is motivated, in part, by the desire to repair status and power relations in society [6, 7]. Norm violations convey disrespect and allow offenders to assume power over both the victim and society [8, 9]. This threat to existing status/power relations prompts the desire for punishment, which degrades the offenders’ status and demonstrates their powerlessness, thus repairing the status hierarchy in society [10, 11].

As punishment has the potential to regulate social hierarchies, support for punishment might depend on people’s preferences for social hierarchy or equality. This suggestion aligns with research from social dominance theory, which argues that individuals with a preference for social hierarchy offer greater approval to punitive policies that support “law and order”, because they disproportionately punish non-dominant group members, and therefore work to maintain existing hierarchies [12–14]. Supporting this claim, research has repeatedly found associations between high social dominance orientation (SDO) and endorsements of punitive policies, as well as punitiveness in general [15, 16].

However, when punishment results in the subversion of existing social hierarchies—for example, when perpetrators are dominant group members—hierarchical preferences may be associated with more lenient punishment recommendations. This has been the focus of much feminist writing, which has argued that hierarchical norms of male dominance are responsible for historical inadequacies in punishing male violence against women [17, 18]. This work suggests that the motivation to punish male violence is weak in male-dominated cultures, because a key purpose of punishment is to promote stability by maintaining existing status hierarchies, which typically favor men [19, 20]. These arguments align with retributive justice theories of punishment, which would predict that patriarchal norms of male dominance would encourage minimal penalties for male violence, given punishment is aimed at diminishing the power/status of the offender and expressing moral condemnation [6, 11]. Examining this theory at an individual level, we thus hypothesize that individuals who endorse patriarchy-enhancing ideologies will recommend relatively lenient punishment for male perpetrators of sexual harassment against women, compared to those who aim to subvert hierarchical gender relations.

Congruent with the theories above, Manne [20] argues that the desire to preserve social hierarchies motivates leniency towards *high-status* men who perform sexual violence. Manne dubs this phenomenon ‘himpathy’, and states that a key driver of himpathy is a “strong if, typically, unwitting disposition to protect dominant men’s interests” [20] (p. 219). Thus, Manne’s argument suggests that preferences for hierarchical relations should predict leniency for high-status perpetrators, specifically. This theory dovetails with research finding that perpetrator status interacts with hierarchical preferences to predict punishment recommendations for other, non-sexual crimes. For example, an elegant study by Redford and Ratliff [5] found that high levels of SDO predicted greater support for retribution in response to a physical assault, though only when the offender was described as low in social status. Other research has found that associations between SDO and punitiveness are significantly stronger when an offender is described as African American as opposed to White, or when punishment is designed to maintain power over criminal offenders [21, 22]. Conversely, ‘egalitarians’ (i.e., those against social hierarchies) are less likely to punish those of low social status (i.e., immigrants and blue-collar workers) when compared to those of high social status, suggesting that the motivation to attenuate hierarchical structures can also induce differential punitive treatment [4, 5].

Taken together, these findings suggest that people differentially punish high and low status individuals to make society conform more closely to their hierarchical preferences. We therefore predict that individuals who want to maintain hierarchical relations will recommend lenient punishment to high (vs. low) status perpetrators of sexual harassment and that the reverse will be true for those who prefer egalitarianism.

### Perpetrator status

The existing literature offers conflicting theories about the direct effect of perpetrator status on punishment recommendations for sexual harassment. Sociological research suggests harassment from high-status perpetrators may be overlooked, as high-status offenders are theorized to have greater ‘moral credentials’ [23]. Consequently, their bad behavior is seen as less wrong and punished with greater moderation [24]. Supporting this prediction, coercive sexual advances are judged as more acceptable when perpetrators are of high socioeconomic status [25] and perceiving an accused rapist as ‘successful’ is associated with more lenient moral judgments [26]. Yet, recent psychological research suggests that individuals with greater social status should be perceived as more ‘morally agentic’—intentional, blameworthy, and causally responsible for their actions [27, 28]. Consistent with this reasoning, an experimental study by Fragale, Rosen, Xu and Merideth [29] found that observers attributed greater intentionality to high (vs. low) status tax avoiders, and consequently recommended more severe punishment. This research suggests that, in some cases, high status can be a liability for those who transgress, as they are seen as more responsible and accountable for their actions.

The current study therefore makes no specific hypotheses regarding the main effect of perpetrator status on punitive responses to sexual harassment. Instead, we examine whether the ‘morally credentialed’ and ‘morally responsible’ accounts of status can help to explain the predicted interaction of SDO and perpetrator status. To that end, we investigate whether the predicted interaction effect of SDO and perpetrator status is mediated by differences in perceived wrongness and perpetrator moral agency.

### The present research

Applying the ‘punishment-as-hierarchy-regulation’ framework to sexual harassment, we make several novel predictions. First, we predict that individuals who endorse hierarchy-enhancing ideologies—as assessed by SDO and gender system justification (GSJ; desire to maintain the existing gender system)—will recommend more lenient punishment for perpetrators of sexual harassment, whilst individuals who are motivated to attenuate hierarchical sex differences will recommend harsher punishment.

Second, we predict that a preference for hierarchy will interact with perpetrator status to affect punitive reactions to sexual harassment. Because SDO is aimed at maintaining hierarchical structures *in general*, as opposed to the gender hierarchy in particular, individuals high in SDO should be particularly likely to treat high-status perpetrators of sexual harassment with leniency, when compared to low-status perpetrators. However, the reverse should be true for individuals low in SDO, who should be particularly likely to treat low-status perpetrators of sexual harassment with leniency.

Finally, integrating research on hierarchical preferences with the ‘morally credentialed’ and ‘morally responsible’ accounts of status, we predict that the moderating effect of hierarchical preferences and perpetrator status will be mediated by the perceived wrongness and the perceived moral agency of the perpetrator.

We conducted three vignette studies to test these hypotheses. In each study participants read descriptions of sexual harassment perpetrated by either high or low status males and rated the appropriateness of punishment recommendations, the wrongness of the behavior, the moral agency of the perpetrator, and completed measures of GSJ and SDO. Drawing on Berdahl [30] we conceptualize sexual harassment as any ‘‘behavior that derogates, demeans, or humiliates an individual based on that individual’s sex” (p. 664). Subsumed under this umbrella term are three, related categories of behavior [31, 32]. Gender harassment captures behaviors that convey insulting, hostile, and degrading attitudes about women; unwanted sexual attention refers to the expression of romantic or sexual interest that are unwelcome, unreciprocated, and offensive to the recipient; and sexual coercion refers to bribes or threats that make the conditions of the victim’s employment contingent on her sexual cooperation. The vignettes in our studies used modified versions of real-world examples of both gender harassment and unwanted sexual attention (e.g., anecdotes from everydaysexism.com). We did not include instances of sexual coercion in our vignettes, as we predicted that the severity of this form of sexual harassment would lead to ceiling effects for our dependent variables.

Study 1 and 3 utilized vignettes that described workplace harassment, whilst Study 2 used vignettes that described stranger harassment. Additionally, Study 3 measured responses to news articles that described sexual harassment, to examine correlations between hierarchical ideologies and real-world instances of harassment. To succinctly summarize findings across studies, we conducted an integrative data analysis [33] that pooled the data across the three studies.

## Study 1

### Method

#### Participants

A power analysis suggested a sample size of 244 to detect a small-medium interaction effect (*f*^*2*^ = 0.05, power = .80) in a linear multiple regression. Qualtrics was used to recruit 371 Australian participants. Thirty-one participants were excluded for failing attention checks, leaving a final sample of 340 (49% male, aged 18–83, *M* = 44.00, *SD* = 18.20). All studies were approved by the Human Ethics Advisory Group at the University of Melbourne (Ethics approval numbers: 13817, 14528, 20859); informed, written consent was obtained online from participants before they completed the surveys.

#### Materials and procedure

All participants viewed three vignettes (see S1 File) that described a male co-worker sexually harassing their female colleague, the order of which was randomized. In line with previous research [34] we manipulated status by modifying the perpetrator’s occupation. Participants were randomly allocated to either the high- or low-status condition; in the high-status condition, participants read that the perpetrators of all three vignettes were Senior Members of the Executive Team. Those in the low-status condition read that the perpetrators were Junior Sales Assistants. To strengthen the status manipulation, we also modified the perpetrator’s residence, locating the high and low status perpetrators in urban and regional areas, respectively, as the latter have greater socioeconomic disadvantage in Australia [35].

#### Measures

After reading each vignette, participants completed two manipulation checks that asked: “how much status do you believe [perpetrator] has in society/within his organization” (1 = very low status; 9 = very high status). We suspected the manipulation might also influence the perceived power inequality between victim and harasser, so we asked participants to indicate “how much power do you believe [perpetrator] has over [victim]” (1 = no power whatsoever; 9 = complete power), as a control variable, as prior research has found that individuals judge harassment more harshly when there is a power inequality between the harasser and target [36, 37].

Participants also completed Effron and Monin’s [38] four-item measure of wrongness (e.g., “to what extent do you find [perpetrator’s] behaviour, 1 = perfectly okay, 9 = extremely immoral”), and indicated their perceptions of the perpetrator’s moral agency, by completing Chan and Haslam’s [39] five items measure (e.g., “how deliberate was [perpetrator’s] behaviour”, 1 = not intentional at all, 9 = completely intentional) in response to each vignette. Participants also completed Bowles and Gelfand’s [34] five-item measure of punishment (e.g., “to what extent do you agree that [perpetrator’s] behaviour should be punished”, 1 = completely disagree, 9 = completely agree) in response to each vignette.

Participants then completed Jost and Kay’s [40] 8-item measure of gender system justification (e.g., “in general, relations between men and women are fair” (1 = completely disagree; 9 = completely agree) and Pratto et al.’s [16] 16-item social dominance orientation scale (e.g., “It’s probably a good thing that certain groups are at the top and other groups are at the bottom” (1 = completely disagree; 7 = completely agree), as well as demographic information including age, gender, and political orientation. Overall indexes for scales were calculated by taking the mean of all items after reverse coding.

### Results

Scale means averaged across vignettes and between-subject reliabilities are presented in Table 1. All scales had very good internal reliability. One outlier was excluded as their Mahalanobis distance statistic fell below the alpha criterion of *p* = .001. However, we draw identical statistical conclusions when including the participant in the analyses. Data and code for all studies are available on the Open Science Framework (https://osf.io/qf79e/?view_only=2c3565e7b165468f918400bb7943f8a1).

**Table 1:** Scale means and between-subject reliabilities from Study 1.

Variable	α	Range	M	SD
Punishment	0.93	1–9	5.72	1.46
Wrongness	0.96	1–9	6.50	1.45
Agency	0.91	1–9	6.49	1.52
GSJ	0.87	1–9	5.49	1.39
SDO	0.93	1–7	2.52	1.14

#### Manipulation checks

Averaging across vignettes, Welch’s t-tests confirmed that participants in the higher status condition saw the perpetrator as significantly higher in general status (*M* = 5.62) and organizational status (*M* = 6.41) than those in the low status condition (*M* = 4.58; *M* = 4.60), *t*(1,332) = 6.00, *p* < .001, *t*(1,338) = 9.88, *p* < .001.

#### Punishment

**Fig 1:**
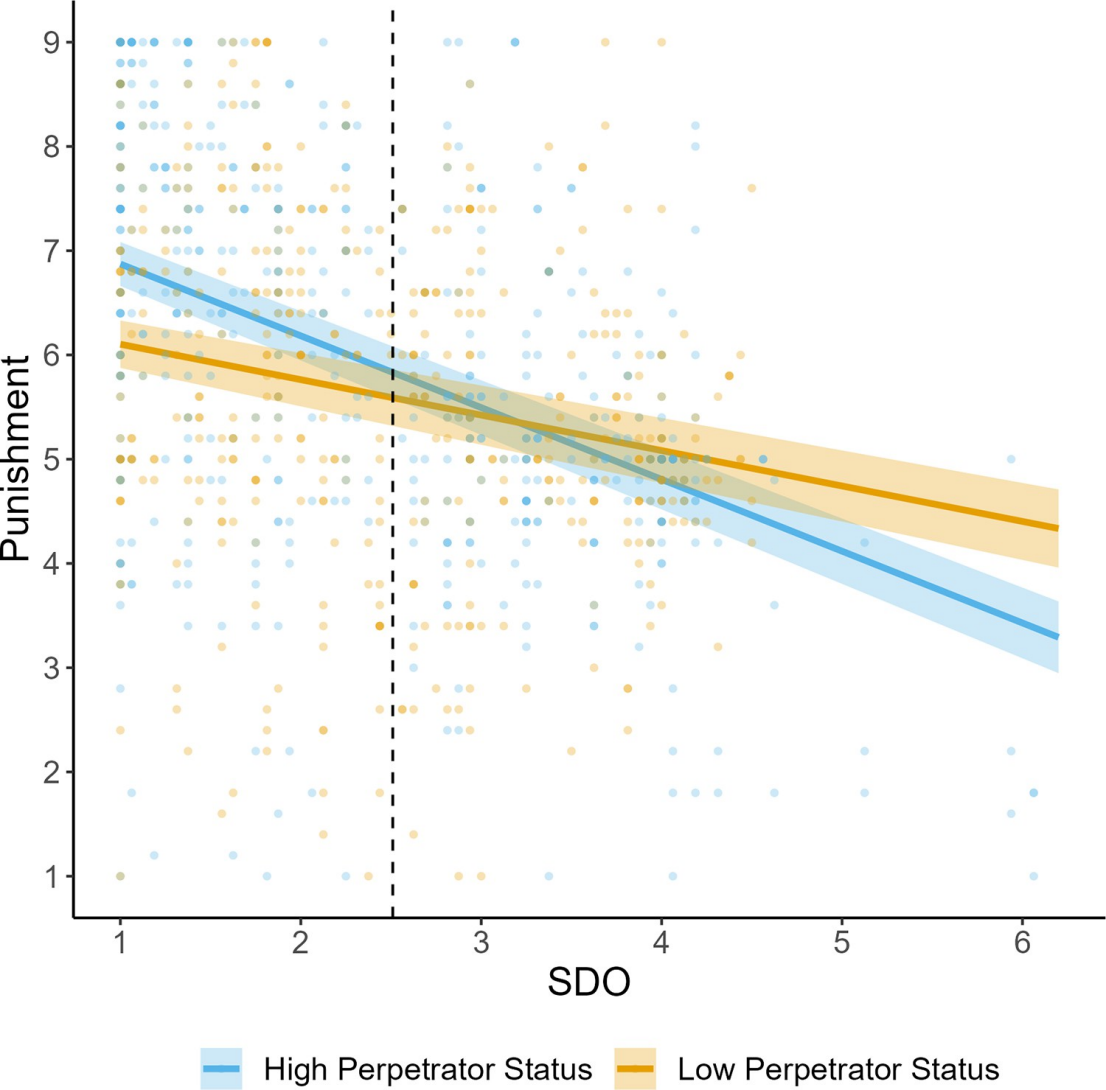
Study 1 punishment judgments as a function of perpetrator status and social dominance orientation. *Note*. Shaded areas display standard error bands. Horizontal dashed line displays the mean score of SDO.

A linear mixed-effects model predicted punishment recommendations from status, GSJ, SDO, and the interaction of status and SDO (see Table 2 and Fig 1). We included a random intercept for participant and vignette to account for the within-subject nature of the dependent variables, and to control for the random effect of vignette on the dependent variables. For this and all future models, status was dummy coded (-0.5 = low status, 0.5 = high status) and SDO was mean-centred. Results demonstrated a marginal effect of status on punishment, whereby participants provided marginally more severe punishment recommendations to high (vs. low) status perpetrators. As predicted, both SDO and GSJ were associated with significantly more lenient punishment. Moreover, SDO and status interacted to significantly predict perpetrator punishment. Findings did not vary substantively when including perceived power inequality, gender, political orientation, or age as control variables. To view models that include control variables and outliers, see S1 File.

**Table 2:** Multilevel regression predicting perpetrator punishment from status, SDO, GSJ, and the interaction of SDO and status.

				95% CI	
	*b*	*SE*	*p*	lower	upper
**Status**	0.24^†^	0.13	.066	-0.02	0.50
**SDO**	-0.51***	0.06	< .001	-0.63	-0.39
**GSJ**	-0.24***	0.05	< .001	-0.34	-0.15
**SDO x Status**	-0.35**	0.12	.004	-0.58	-0.11
**Random effects**					
**σ2**	1.40				
**τ00 ID**	1.00				
**τ00 vignette**	0.07				
Marginal R^2^	.21				
Conditional R^2^	.55				

*Note*.

^†^*p* < .10

*p < .05

**p < .01

***p < .001. Under random effects, σ2 is the residual variance of the model. τ00 parameters are the random intercept variances for participant. CI = confidence interval.

Simple effects analyses examining the interaction of SDO and status revealed that at high levels of SDO (+1SD) there was no effect of status on perceived punishment *b* = -0.15, *SE* = 0.19, *t*(334) = -0.80, *p* = .42. However, at low levels of SDO (-1SD), participants recommended significantly harsher punishment to high (vs. low) status perpetrators *b* = 0.64, *SE* = 0.19, *t*(334) = 3.36, *p* < .001.

Johnson-Neyman Intervals revealed that participants with SDO scores below 2.45 (on a 7-point scale: -0.06 SDs) were estimated to provide more lenient punishment recommendations to low status perpetrators, whereas participants with scores above 5.07 (+2.24 SDs) were estimated to provide more lenient punishment recommendations to high status perpetrators.

#### Moderated mediation

We tested whether the interaction effect of SDO and perpetrator status was mediated by perceptions of wrongness and moral agency using the PROCESS macro in SPSS. We predicted that both variables would partially account for the interaction effect. To test these hypotheses, we fit moderated mediation model 8 with 5,000 bootstrapped resamples [41]. As this macro cannot analyze multilevel data, we created composite scores that averaged across vignettes.

**Fig 2:**
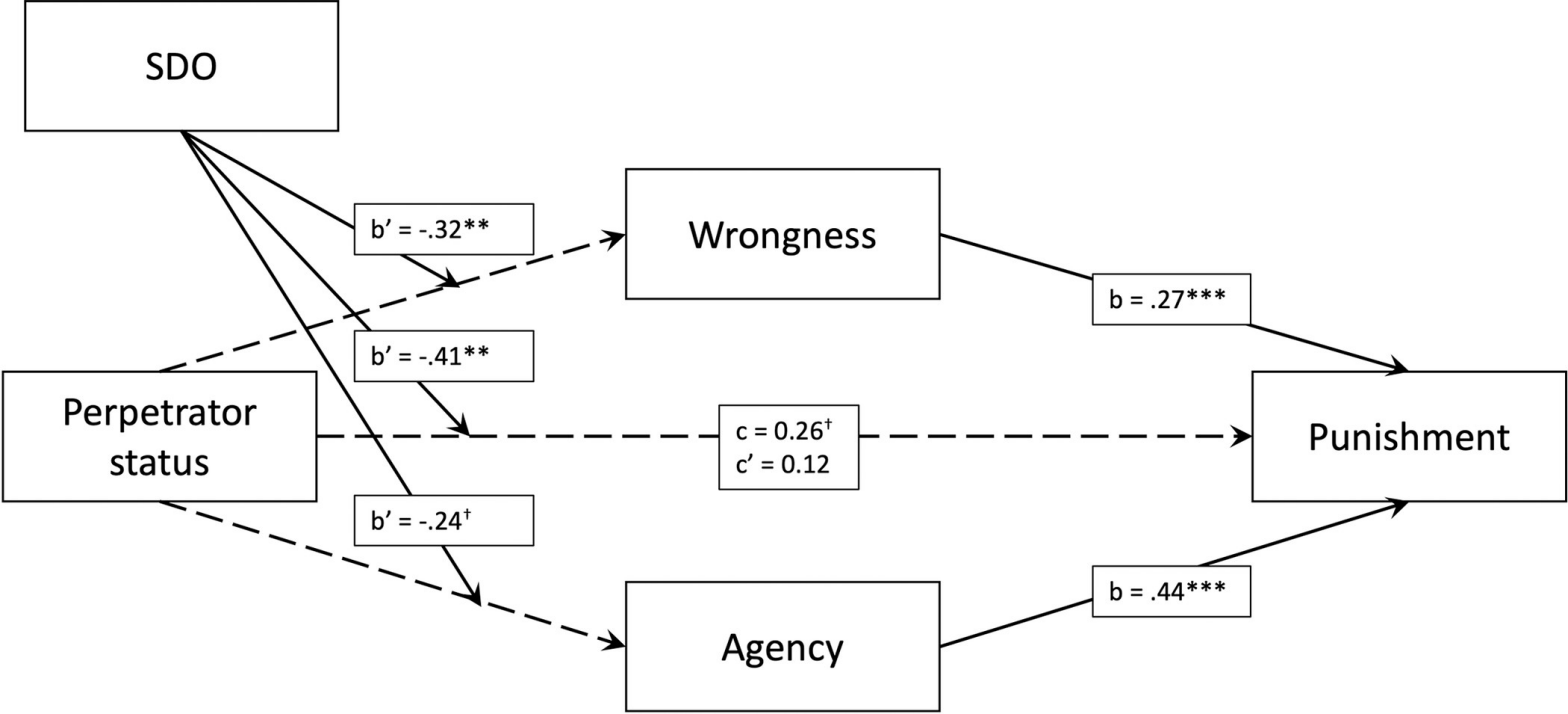
Moderated mediation model, b’ specifies interaction coefficients. *Note*. ^†^*p* < .10, *p < .05, **p < .01, ***p < .001.

Bootstrapped moderated mediation analyses demonstrated that the interaction effect of perpetrator status and SDO on punishment was significantly mediated by wrongness, (*b* = -0.09, BootSE = 0.04, BootCIs (-0.18, -0.02)), but not moral agency (*b* = 0.10, BootSE = 0.07, BootCIs (-0.25, 0.01)) (see Fig 2). For those high on SDO (+1SD), there was no significant indirect effect of status on punishment via moral wrongness (*b* = -0.07, BootSE = 0.06, BootCIs (-0.19, 0.04)). However, there was a significant indirect effect of status on punishment via moral wrongness for those with low levels of SDO (-1SD), (*b* = 0.13, BootSE = 0.06, BootCIs (0.02, 0.27)). When including the control variables, the overall mediation models were statistically identical. However, the indirect effect of status on punishment via moral wrongness for those with low levels of SDO became non-significant (*b* = 0.08, BootSE = 0.06, BootCIs (-0.02, 0.20)).

### Discussion

Study 1 provides support for our two key hypotheses. SDO and GSJ independently predicted punishment recommendations for sexual harassment, where greater endorsement of these hierarchy-enhancing ideologies was associated with milder punishment recommendations for perpetrators. Additionally, SDO interacted with perpetrator status to predict punishment recommendations. This effect was primarily driven by those low on SDO, who provided harsher punishment recommendations for perpetrators of high (vs. low) status. Surprisingly, individuals who scored relatively high on SDO did not differentiate between high and low status perpetrators. Only individuals with absolutely high scores on SDO (above 5.07 on a 7-point scale; in the 98th percentile) were estimated to provide more lenient punishment recommendations to high (vs. low) status perpetrators. Taken together, these results provide evidence for our central assertion that individuals strategically punish sexual harassment in a way that supports their preference to maintain or eliminate hierarchical structures. However, the findings suggest that only egalitarians and statistically extreme inegalitarians are prone to use selective punishment to differentiate between high and low status perpetrators.

Unexpectedly, participants made marginally more severe punishment recommendations for high vs. low status perpetrators. This is consistent with the ‘morally responsible’ account of status, which argues that higher status wrongdoers are treated with greater severity due to higher perceived moral agency. However, our moderated mediation analyses suggested that the interaction effect was mediated by wrongness perceptions, not perceptions of moral agency. Thus, our data are consistent with a model in which individuals’ differing punishment recommendations due to perpetrator status and SDO are explained by varying perceptions of how wrong the sexual harassment is.

## Study 2

Study 2 aimed to replicate the findings from Study 1 using three vignettes describing stranger rather than workplace harassment. The stranger harassment context allows a test of generalizability and allows status to be manipulated independent of power: regardless of status, the unknown perpetrator has no power to affect the victim’s future employment. This manipulation does not remove the *gendered* status and power imbalances between the male perpetrators and female targets [30], but instead ensures that the high- and low- status perpetrators do not have unequal power over their female targets. Hypotheses and analyses were preregistered using www.aspredicted.org (see S1 File).

### Method

#### Participants

Qualtrics was used to recruit 378 Australian participants, but two participants were excluded for failing attention checks, leaving a final sample of 376 (51% male, aged 18–87, *M* = 49.90, *SD* = 18.10).

#### Materials and procedure

Materials, procedures, and methods were identical to those in the first study, with the exception of the three vignettes, which described stranger harassment (see S1 File). Participants were shown all three vignettes describing stranger harassment. The order in which they were displayed was randomized. Like Study 1, perpetrator status was manipulated by varying occupation (Senior Executive within a large corporation *vs*. a tradesperson, truckdriver, or cleaner).

### Results

Scale means averaged across vignettes and between-subjects reliabilities are presented in Table 3. As with Study 1, all scales demonstrated very good reliability. No observations were identified as outliers using the preregistered criterion: a Mahalanobis distance statistic *p* < .001.

**Table 3:** Scale means and between-subject reliabilities from Study 2.

Variable	α	Range	M	SD
Punishment	0.93	1–9	5.30	1.46
Wrongness	0.97	1–9	6.52	1.46
Agency	0.95	1–9	6.44	1.47
GSJ	0.83	1–9	5.62	1.42
SDO	0.93	1–7	2.53	1.11

#### Manipulation checks

Welch’s t-tests confirmed that participants in the higher status condition saw the perpetrator as significantly higher in general status (*M* = 5.67) and organizational status (*M* = 6.67) than those in the low status condition (*M* = 4.25; *M* = 4.17), *t*(1,367) = 8.29, *p* < .001, *t*(1,370) = 14.08, *p* < .001.

#### Punishment

**Fig 3:**
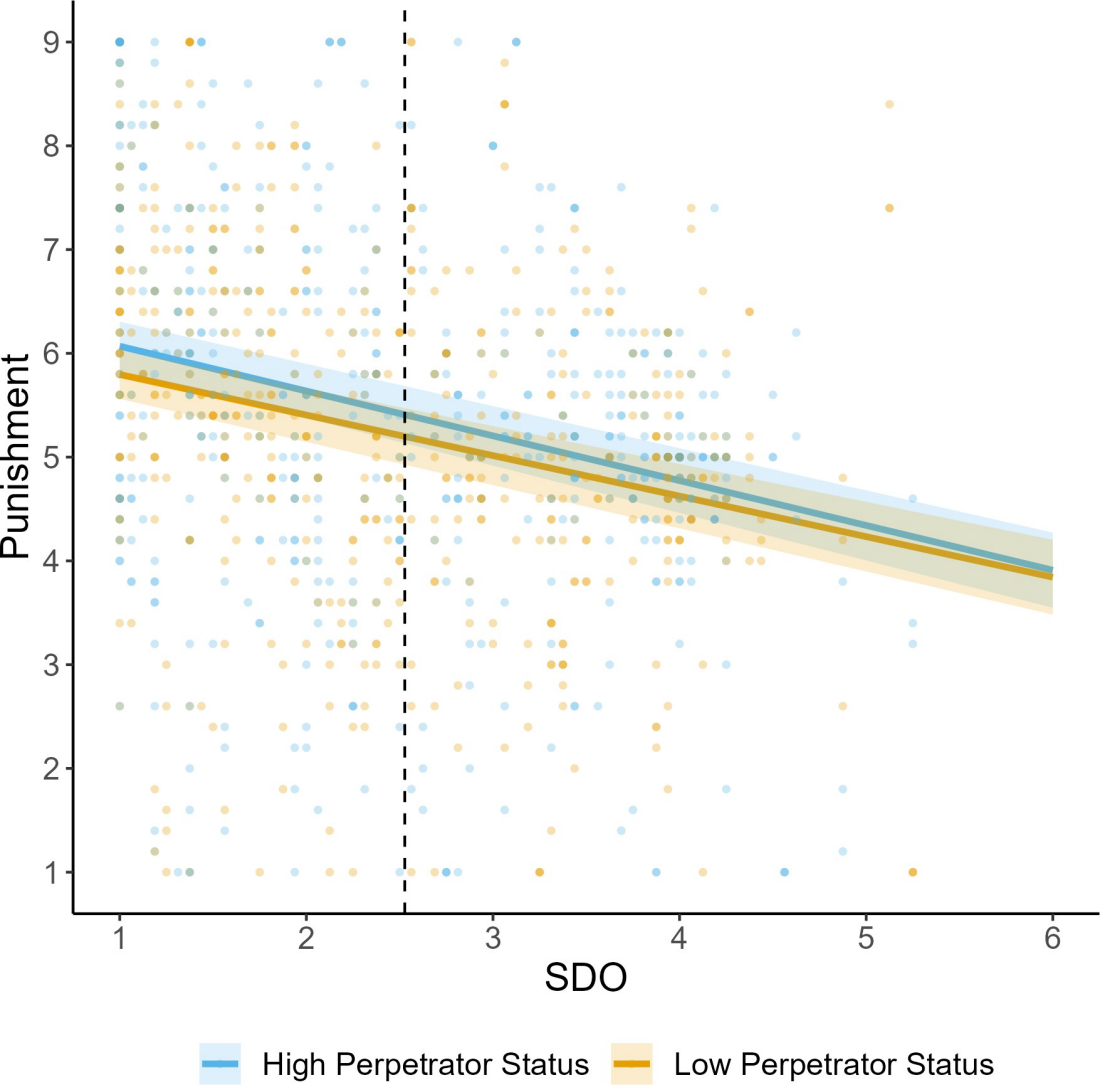
Study 2 punishment judgments as a function of perpetrator status and social dominance orientation. *Note*. Shaded areas display standard error bands. Horizontal dashed line displays the mean score of SDO.

We fit a linear mixed-effects model with a random intercept for participant and vignette and fixed factors of status, GSJ, SDO, and the interaction terms of status and SDO (see Table 4 and Fig 3). There was no significant effect of status on punishment. As predicted, both SDO and GSJ predicted more lenient punishment recommendations. However, the interaction of SDO and status was not significant. Findings do not change substantively when including perceived power inequality, gender, political orientation, or age as control variables.

**Table 4:** Study 2 multilevel regression predicting perpetrator punishment from status, SDO, GSJ, and the interaction of SDO and status.

				95% CI	
	*b*	*SE*	*p*	lower	upper
**Status**	0.21	0.14	.128	-0.06	0.48
**SDO**	-0.41***	0.06	< .001	-0.53	-0.29
**GSJ**	-0.23***	0.05	< .001	-0.33	-0.13
**SDO x Status**	-0.04	0.12	.740	-0.28	0.20
**Random Effects**					
**σ2**	1.35				
**τ00 ID**	1.32				
**τ00 vignette**	0.08				
**Marginal R^2^**	0.12				
**Conditional R^2^**	0.57				

Note.

*p < .05

**p < .01

***p < .001. Under random effects, σ2 is the residual variance of the model. τ00 parameters are the random intercept variances for participant. CI = confidence interval.

### Discussion

Study 2 provides partial support for our hypotheses. As in Study 1, higher SDO and GSJ independently predicted more lenient punishment recommendations for perpetrators of sexual harassment. However, we were unable to replicate the interaction effect of SDO and perpetrator status. There are several factors that could explain the discrepant interaction effects between studies. The vignettes used in Study 2 described the low-status perpetrators as a truckdriver, tradesperson, or cleaner, as opposed to a junior sales assistant, as in Study 1. Given Study 1’s interaction effect was predominantly driven by egalitarians, who provided more lenient recommendations to low-status perpetrators, Study 2 may have uncovered a boundary condition for their leniency. Prior research has shown that ‘educationism’—prejudice against those with low levels of education—is a widespread and potent phenomenon [42]. Indeed, negative views towards the uneducated are held by individuals often perceived to be most egalitarian: the highly educated [43]. Thus, the discrepancy between studies may indicate that egalitarians are willing to provide more lenient punishment for educated low-status individuals, as in Study 1, but not to those who lack a formal education, as in Study 2.

It is also possible that egalitarians are unwilling to provide any lenient punishment (even for low-status perpetrators) when responding to stranger-harassment, as it elicits stronger negative reactions when compared to harassment from a co-worker or an acquaintance [44, 45]. Alternatively, the absence of a perpetrator power differential across the status conditions in Study 2 may be implicated, although this is unlikely because controlling for power did not eliminate the significant interaction in Study 1. The specific reason for the non-replication is therefore unclear.

## Study 3

Study 3 aimed to clarify the conflicting results regarding the interaction of perpetrator status and SDO across the previous two studies. Our primary aim was to establish whether the original interaction effect was robust in a direct replication, so we utilized the same vignettes as in Study 1. Additionally, we extended our previous studies by also examining punishment recommendations in response to news articles that described real-world instances of sexual harassment by high-status men, to determine whether hierarchical preferences predict reactions to real-world instances of sexual harassment. Hypotheses and analyses were preregistered using www.aspredicted.org (see S1 File).

### Method

#### Participants

Qualtrics was used to recruit 570 Australian participants. We excluded 16 participants for failing attention checks, leaving a final sample of 554 (48% male, aged 18–86, *M* = 38.10, *SD* = 16.60).

#### Materials and procedure

All participants first read and responded to the three vignettes describing workplace harassment used in Study 1. Subsequently, they responded to three news articles describing real-world instances of sexual harassment. Procedures were identical to those in the first study, other than the inclusion of news articles summarizing instances of sexual harassment committed by high-status Australian men, following the vignettes. All news articles noted that the perpetrator resigned from their position after an investigation confirmed they had behaved inappropriately. After reading each article, participants rated whether they agreed the perpetrator should no longer be in his role (1 = strongly disagree; 10 = strongly agree), and the severity of punishment they should receive (1 = not severe at all; 10 = extremely severe). These scores were used to create a composite punishment score. We also asked participants how familiar they were with the allegation (1 = not familiar at all; 9 = extremely familiar) to use as a control variable.

### Results

Scale means averaged across vignettes and between-subjects reliabilities are presented in Table 5. Five participants whose Mahalanobis distance statistic fell below the pre-registered alpha criterion of *p* = .001 were excluded from the analyses as outliers.

**Table 5:** Scale means and between-subjects reliabilities from Study 3.

Variable	α	Range	M	SD
Punishment	0.94	1–9	5.84	1.56
Wrongness	0.97	1–9	6.88	1.44
Agency	0.96	1–9	6.86	1.51
Punishment (News)	0.92	1–10	7.47	1.76
GSJ	0.83	1–9	5.30	1.41
SDO	0.93	1–7	2.38	1.09

#### Manipulation checks

Averaging across vignettes, Welch’s t-tests confirmed that participants in the higher status condition saw the perpetrator as significantly higher in general status (*M* = 6.09) and organizational status (*M* = 6.95) than those in the low status condition (*M* = 4.38; *M* = 4.14), *t*(1,539) = 11.78, *p* < .001, *t*(1,518) = 18.98, *p* < .001.

#### Punishment

**Fig 4:**
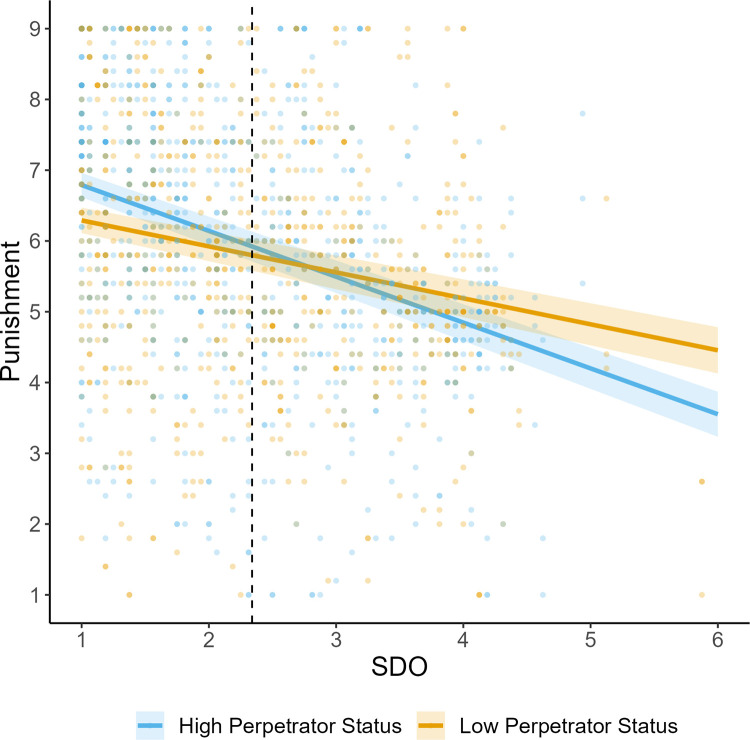
Study 3 punishment judgments as a function of perpetrator status and social dominance orientation. *Note*. Shaded areas display standard error bands. Horizontal dashed line displays the mean score of SDO.

We conducted linear mixed-effects models with a random intercept for participant and vignette, and fixed factors of status, GSJ, SDO, and the interaction terms of status and SDO (see Table 6 and Fig 4). There was no significant effect of perpetrator status on punishment. As hypothesized, both SDO and GSJ predicted milder punishment recommendations. The predicted interaction effect of SDO and status was also obtained, but weakened when outliers were included (*b =* 0.19, SE = 0.11, *p* = .07). No effects varied substantively when including outliers, nor when including perceived power inequality, gender, age, or political orientation as control variables. To view models that include control variables and outliers, see S1 File.

**Table 6:** Study 3 multilevel regression predicting perpetrator punishment from status, SDO, GSJ, and the interaction of SDO and status.

					95% CI	
	*b*	*SE*		*p*	lower	upper
**Status**	0.12	0.11		.318	-0.11	0.33
**SDO**	-0.51***	0.06		< .001	-0.62	-0.40
**GSJ**	-0.31***	0.04		< .001	-0.39	-0.23
**SDO x Status**	-0.28*	0.11		.011	-0.50	-0.06
**Random Effects**						
**σ2**	1.46					
**τ00 ID**	1.25					
**τ00 vignette**	0.04					
**Marginal R^2^**	0.18					
**Conditional R^2^**	0.57					

*Note*.

*p < .05

**p < .01

***p < .001. Under random effects, σ2 is the residual variance of the model. τ00 parameters are the random intercept variances for participant. CI = confidence interval.

Simple effects analyses examining the interaction of SDO and status revealed that at high levels of SDO (+1SD) there was no effect of status on perceived punishment *b* = -0.16, *SE* = 0.16, *t*(544) = -1.02, *p* = .31. However, at low levels of SDO (-1SD), there was a significant effect of status on punishment *b* = 0.41, *SE* = 0.16, *t*(544) = 2.56, *p* = .01, where participants were more likely to punish high (vs. low) status perpetrators.

Johnson-Neyman Intervals revealed that participants with SDO scores below 1.93 (on a 7-point scale: -0.41 SDs) were estimated to provide more lenient punishment recommendations to low status perpetrators, whilst participants with scores above 4.92 (+2.33 SDs) were estimated to provide more lenient punishment recommendations to high status perpetrators.

#### Moderated mediation

We tested whether the interaction effect was mediated by perceptions of moral wrongness and moral agency. The procedure was identical to Study 1. Bootstrapped moderated mediation analyses demonstrated that the moderation effect of perpetrator status and SDO on punishment was neither mediated by perceived wrongness (*b* = -0.02, BootSE = 0.03, BootCIs (-0.08, 0.04)), nor perpetrator moral agency (*b* = -0.05, BootSE = 0.06, BootCIs (-0.18, 0.06)).

#### Punishment–news articles

We fit a linear mixed-effects model that predicted punishment recommendations for the perpetrators of sexual harassment discussed in the news articles. We included random intercepts for participant and article, as well as fixed factors of GSJ and SDO. As predicted, both variables independently predicted punishment recommendations (see Table 7). Findings do not change substantively when including case familiarity, gender, age, or political orientation as control variables.

**Table 7:** Study 3 multilevel regression predicting news-article perpetrator punishment from SDO and GSJ.

				95% CI	
	*b*	*SE*	*p*	lower	upper
**SDO**	-0.39***	0.07	< .001	-0.53	-0.26
**GSJ**	-0.16**	0.05	.002	-0.26	-0.06
**Random Effects**					
**σ2**	1.75				
**τ00 ID**	2.00				
**τ00 article**	0.09				
Marginal R^2^	0.06				
Conditional R^2^	0.57				

*Note*.

*p < .05

**p < .01

***p < .001. Under random effects, σ2 is the residual variance of the model. τ00 parameters are the random intercept variances for participant. CI = confidence interval.

### Discussion

Replicating and extending our previous findings, Study 3 found that both SDO and GSJ predicted milder punishment recommendations for perpetrators of sexual harassment, in response to both hypothetical scenarios and real-world instances of sexual harassment. Additionally, the results demonstrate an interaction effect of SDO and perpetrator status, replicating Study 1. Simple effects analyses replicated the surprising asymmetry in selective punishment found in Study 1, which showed that egalitarians recommended greater punishment to high (vs. low) status perpetrators, but individuals who scored relatively high on SDO did not differentiate between the two perpetrators. Only individuals with absolutely high scores on SDO (above 4.92 on a 7-point scale; i.e., at or above the 98th percentile) were estimated to provide more lenient punishment recommendations to high (vs. low) status perpetrators.

## Integrative data analysis

We conducted an analysis that combined the three studies to estimate the overall magnitude of the effects. In contrast to a mini meta-analysis, we opted for an integrative data analysis that pools the raw data from all studies [33]. This approach avoids extracting effect sizes for multilevel models, which lack a unified methodology in existing literature [46, 47].

We used a multilevel model with participants (*N* = 1270) nested within studies, treating studies, vignettes, and participants as random factors. Conceptually, the multilevel approach allows us to examine the effect of our predictors, taking account of random variation due to participants, vignette, and study. We opted for a Bayesian approach to estimating the model, which is recommended by Brauer and Curtin [48] and has been successfully employed in previous psychological research [49]. The stan_lmer() function of the rstanarm package was used to fit the model, and we used the function’s default, adjusted, weakly informative priors: a normal distribution with mean 0 and standard deviation 4.5—symbolized as N(0, 4.5)—for the intercept, N(0, 4.2), N(0, 9.0), N(0, 3.2), and N(0, 8.3) for status, SDO, GSJ and the SDO and status interaction, respectively.

### Results

Table 8 reports the estimates and 95% posterior intervals for model parameters. Diagnostic checks [50] found no divergent transitions and R-hat never exceeded 1.1, indicating satisfactory convergence. We concluded there was evidence for an effect whenever the 95% credibility interval excluded zero.

**Table 8:** Multilevel Bayesian linear model investigating status, SDO, GSJ and the interaction of SDO and status as predictors of punishment recommendations for sexual harassment vignettes across three studies.

			95% CI	
	Estimates	SE	Lower	Upper
**Status**	0.19	0.08	0.04	0.34
**SDO**	-0.48	0.04	-0.57	-0.39
**GSJ**	-0.27	0.03	-0.34	-0.20
**SDO x Status**	-0.22	0.07	-0.36	-0.07
**Random Effects**				
σ^2^	1.41			
**τ00 participant**	1.21			
**τ00 study**	<0.01			
**τ00 vignette**	0.20			
**τ11 study.SDO**	<0.01			
**τ11 study.status**	<0.01			
**τ11 study.GSJ**	<0.01			
**τ11 study.SDO x status**	<0.01			
**Marginal R^2^**	0.16			
**Conditional R^2^**	0.59			

*Note*. Under random effects, σ2 is the residual variance of the model. τ00 parameters are the random intercept variances for participant, study, and vignette, τ11 are the random slope variances for the predictors. CI = credibility intervals

**Fig 5:**
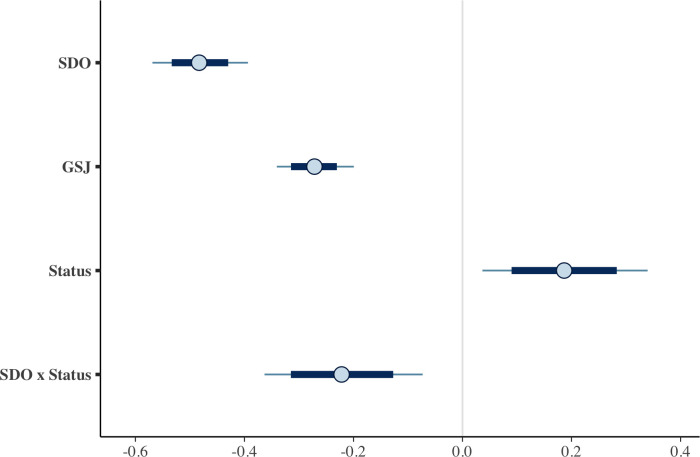
Plot of estimates of regression coefficients for all predictors. *Note*. The circle indicates the median (quantile-based) posterior Bayesian estimates, the heavy line indicates the 80% credible interval, and the thin line indicates the 95% credible interval.

As expected, SDO and GSJ were associated with more lenient punishment recommendations (see Fig 5). There was a modest interaction between SDO and status, where the effect of SDO was reduced by 0.22 when evaluating low-status perpetrators. Additionally, there was a small effect of perpetrator status on punishment recommendations, where those of high status received slightly more severe punishment recommendations. However, credibility intervals for this effect included zero in the model that included relevant control variables (perceived power inequality, gender, age, and political orientation). All other effects remained substantively similar in the model that included control variables.

Simple effects analyses examining the interaction of SDO and status revealed that at high levels of SDO (+1SD) there was no effect of status on perceived punishment, *b =* -0.05, 95% CI(-0.25, 0.18). However, at low levels of SDO (-1SD), there was a significant effect of status on punishment *b* = 0.43, 95% CI(0.20, 0.63), whereby participants were more likely to punish high status perpetrators.

## General discussion

Over three studies, we found that greater endorsement of two hierarchy-enhancing ideologies (GSJ and SDO) consistently predicted more lenient punishment recommendations for perpetrators of sexual harassment, in both fictional scenarios and when evaluating real-world news articles. These findings support our hypothesis that individuals with a stronger motivation to uphold the gender hierarchy—because they are invested in maintaining the gender status quo, or are uninterested in changing hierarchical relations—provide milder punishment recommendations for perpetrators of sexual harassment. Additionally, our integrative data analysis uncovered a modest interaction between SDO and perpetrator status, where the negative relationship between SDO and punishment recommendations was stronger when evaluating high (vs. low) status perpetrators. In other words, we found that the tendency for egalitarians to provide harsher punishment recommendations for sexual harassment was greater when evaluating high relative to low status perpetrators. This suggests that egalitarians selectively punish sexual harassment depending on whether perpetrators threaten their preferred level of hierarchy.

Our investigation builds on prior research that suggests individuals use punishment to regulate power relations and reaffirm prized social values [5–8]. Examining this theory in the context of sexual harassment, our studies provide preliminary support that people punish sexual harassment to bolster or attenuate power structures, in keeping with their social hierarchy preferences. These findings may help to explain why opinions about punishing sexual harassment are divided along partisan lines [51], given the strong relationships between hierarchical and political preferences [52, 53].

Unexpectedly, our study revealed that the interaction effect of SDO and perpetrator status on punishment was primarily driven by those low on SDO, who delivered significantly harsher punishments to high status perpetrators. This finding aligns with other, recent research which has found that egalitarians are biased against dominant group members in employment contexts [54]. In tandem, these results provide evidence that egalitarians may not be dispositionally less punitive, as previously thought [15]. Instead, their motivation to punish appears to differ depending on whether the punishment attenuates or enhances social hierarchies.

The asymmetry between high and low SDO participants in their use of selective punishment contributes to a growing body of research indicating that egalitarians, at times, provide stronger differential treatment in favor of disadvantaged group members than high SDO participants do against them [5, 54–56]. It is plausible that egalitarians differentiate on the basis of social status because they are more likely to perceive the status quo as inherently unequal and thus view harsher treatment of high-status perpetrators as a means of reducing inequality. Supporting this theory, research has found that egalitarians are more vigilant for and accurate at detecting inequality than those high on SDO, but only when inequalities affect disadvantaged groups, not when they equivalently affect advantaged groups [57]. It is also possible that because egalitarians tend to view status inequalities as illegitimate and undeserved, they may hold negative stereotypes of high-status individuals as selfish and greedy [58]. If true, these stereotypes could contribute to the harsher penalties bestowed upon high-status perpetrators by egalitarians found in the current studies.

One explanation for why relatively high SDO scorers did not discriminate in favour of high-status perpetrators concerns the low SDO scores typical of our samples. Across our studies, mean levels of SDO ranged from 2.38–2.52 on a 7-point scale, with standard deviations between 1.09–1.14. As such, ‘high’ SDO scorers relative to the sample mean still predominantly sit below the midpoint of the SDO scale, and thus do not in most cases directly endorse social hierarchy, but rather show a greater tolerance or ambivalence towards inequality. Notably, this left-displaced distribution is not anomalous, but is found in samples cross-culturally [59, 60]. Given the normative rejection of social hierarchies, relatively ‘high’ SDO scorers may not explicitly support preferential treatment of high-status group members, but instead deny that inequalities exist [57], or oppose behaviors that lead to greater equality [61]. Supporting this theory, our analyses revealed that participants with *absolute* high levels of SDO (above the scale mid-point) were estimated to provide more lenient punishment recommendations to high (vs. low) status perpetrators. However, only a small fraction of participants sat above this threshold. To thoroughly examine whether an endorsement of social hierarchy results in more lenient punishment recommendations for high-status perpetrators, future research should oversample for high SDO participants.

We found no evidence that individuals recommend harsher punishment for lower status perpetrators, and indeed found some evidence for the opposite effect. This is inconsistent with theories speculating a generalized bias that favors high-status men who perform sexual violence, including Kate Manne’s claim of ‘himpathy’ [18]. Further contrasting Kate Manne’s claims, we did not find strong evidence that individuals with a preference for hierarchy recommend more lenient punishment to high-status perpetrators. Thus, any bias that exists in favor of high-status perpetrators is unlikely to be explained by the desire to preserve social hierarchies. Although high-status men may receive lenient sentences in legal contexts (e.g., the Brock Turner phenomenon [62]), our research suggests these judgments do not reflect a generalized tendency, but may instead reflect a bias specific to the judicial system—a field dominated by high-status men, who may sympathize more with men who resemble themselves [63]. Similarly, organizations may hold biases in favor of ‘valuable’ high-status perpetrators who bring significant revenue for the company, though our research suggests this is unlikely to be caused by a desire to protect social hierarchies, but instead may be driven by economic interests [64]. Our findings also contradict older research which suggest a bias against low-status perpetrators of sexual violence [26, 65]. These differing results may reflect a recent loss of sympathy for high-status perpetrators, potentially caused by the #MeToo movement.

Finally, our findings provide minimal evidence that judgments of wrongness and moral agency explain the interacting effect of SDO and perpetrator status on punishment. Moral agency did not mediate the interaction in any of the studies, and wrongness showed an indirect effect in only one study. Thus, it is unlikely that the differential reactions to high vs. low status perpetrators of sexual harassment from egalitarian and hierarchical participants were due to differences in the perceived moral agency of the perpetrator, or the wrongness of their actions. Future research could examine whether differing empathetic responses to perpetrators better explain the interaction effect, in line with prior research that has identified perpetrator empathy as a key predictor of sexual harassment judgements [66]. Previous research has found that lower levels of SDO predicts greater empathy for nondominant group members who experience hardship [55, 67]. These results support the notion that individuals low on SDO could have had differing empathy for perpetrators of high vs. low status, which may have contributed to their differing punishment recommendations.

Future research should also manipulate the desire to maintain the gender hierarchy, to causally examine its effect on punishment recommendations for sexual violence. In line with retributive justice theories, we propose that the relationship between hierarchical ideologies and punishment reflects differing goals to either maintain or diminish the gender hierarchy [6]. However, because our research is correlational, we cannot rule out the possibility that this relationship simply reflects differing perceptions of how harmful or ‘natural’ sexual violence is. It is also important to highlight that the interaction effect of SDO and perpetrator status appeared inconsistently across our three studies. Although our Bayesian analysis provides evidence that a modest effect exists when consolidating our findings, future research should conceptually replicate our findings with differing vignettes and manipulations, to investigate the generalizability of the effect.

A final limitation of our study concerns the vignettes used in the studies, which only portrayed male perpetration of sexual harassment against women. We focused on male sexual harassment against women as it the most prevalent form of sexual harassment when compared to other gender dyads [68]. However, this focus prevents us from generalizing our findings to all forms of sexual harassment. It is possible that egalitarians might provide equal or more lenient punishment recommendations for female harassment against men, when compared to those high in SDO, as recent work finds egalitarianism predicts greater sympathy for females [69]. To examine this possibility, future research could investigate whether perpetrator gender interacts with SDO to predict punishment recommendations for sexual harassment.

## Conclusion

Understanding discrepant views on punitive responses to sexual harassment is vital in the post #MeToo era, if we want to come to a harmonious understanding on how to deal with perpetrators. In three studies, we found that hierarchy-attenuating ideologies predicted harsher punishment recommendations for sexual harassment, and that this effect was stronger when evaluating high (vs. low) status perpetrators. These findings suggest that individuals punish sexual harassment to bolster or attenuate power structures, depending on their preference for social hierarchy or equality. At a time of heightened global attention on sexual harassment, our work offers a new perspective on why the treatment of perpetrators remains so contentious.

## Supporting information

S1 File Supplemental materials. (DOCX)
