# Supplementary material for: Himpathy and status: Attitudes to social hierarchy predict reactions to sexual harassment
Source: PLoS One. 2023 Dec 6;18(12):e0292953. doi: 10.1371/journal.pone.0292953 (PMC10699639; doi:10.1371/journal.pone.0292953)
Supplement: S1 File — (DOCX) [file pone.0292953.s001.docx]

**Supplemental Materials**

This document contains the following:

| [1. Vignettes used in Study 1 and 3](#_1._Vignettes_used) | Pg. 2 |
| --- | --- |
| [2. Vignettes used in Study 2](#_2._Vignettes_used) | Pg. 5 |
| [3. News articles used in Study 3](#_3._News_Articles) | Pg. 8 |
| [4. OSF and pre-registration links](#_4._OSF_link) | Pg. 11 |
| [5. Study 1 control and outlier analyses](#_5._Study_1) | Pg. 11 |
| [6. Study 2 control analysis](#_6._Study_2) | Pg. 14 |
| [7. Study 3 control and outlier analyses](#_7._Study_3) | Pg. 15 |
| [8. Integrative data analyses with control variables](#_8._Integrative_Data) | Pg. 19 |
| [9. Integrative data analyses with outlier participants](#_9._Integrative_Data) | Pg. 20 |

## 1. Vignettes used in Study 1 and 3


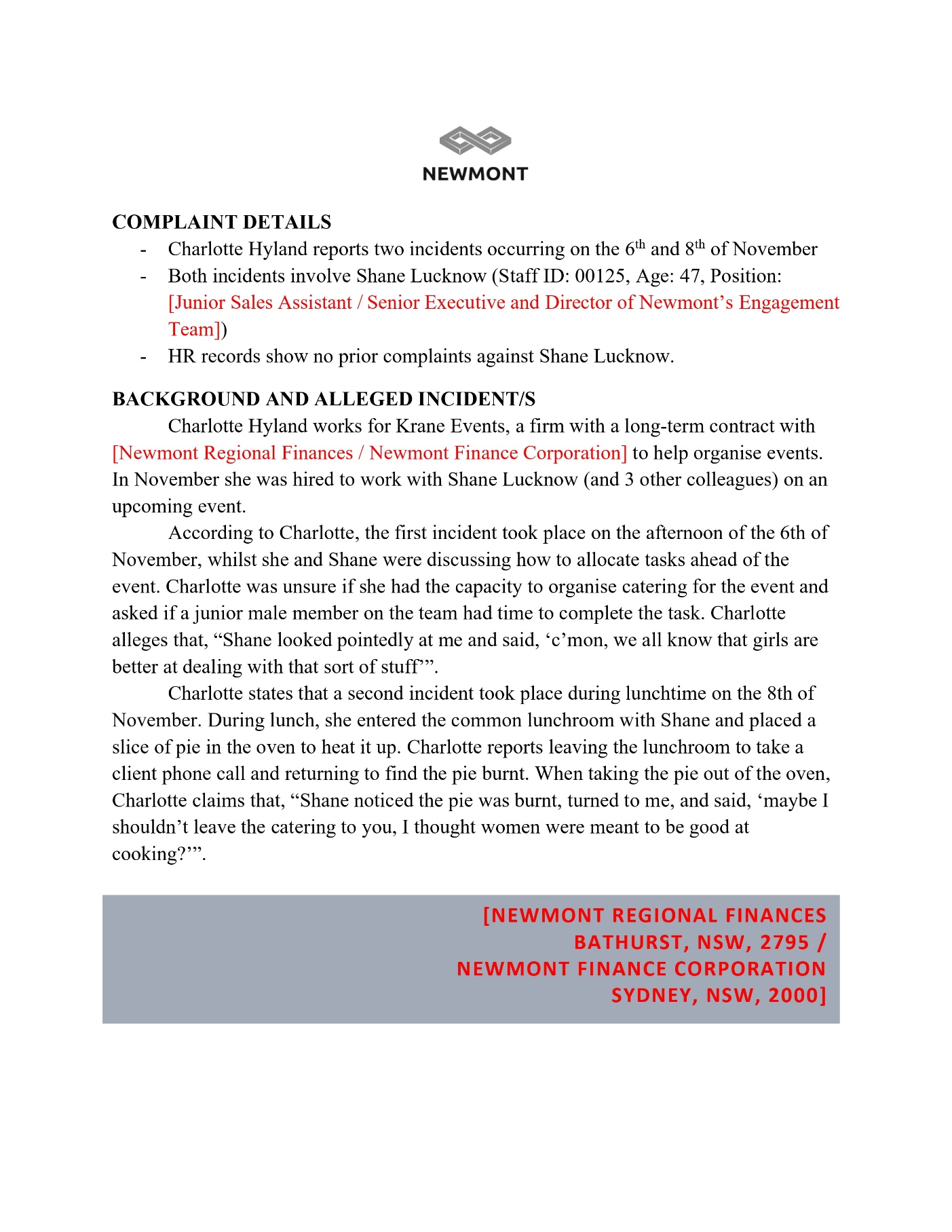


**
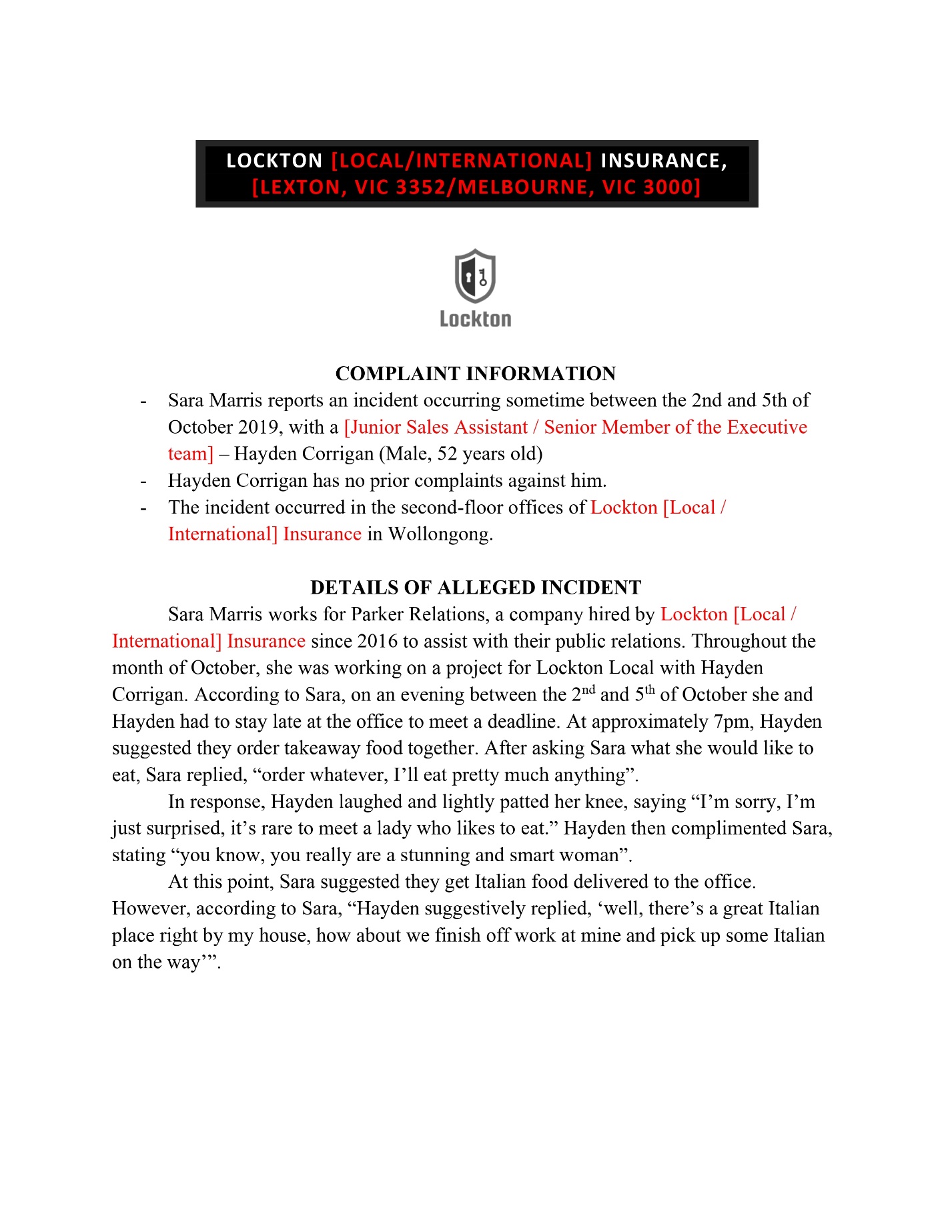
**

**
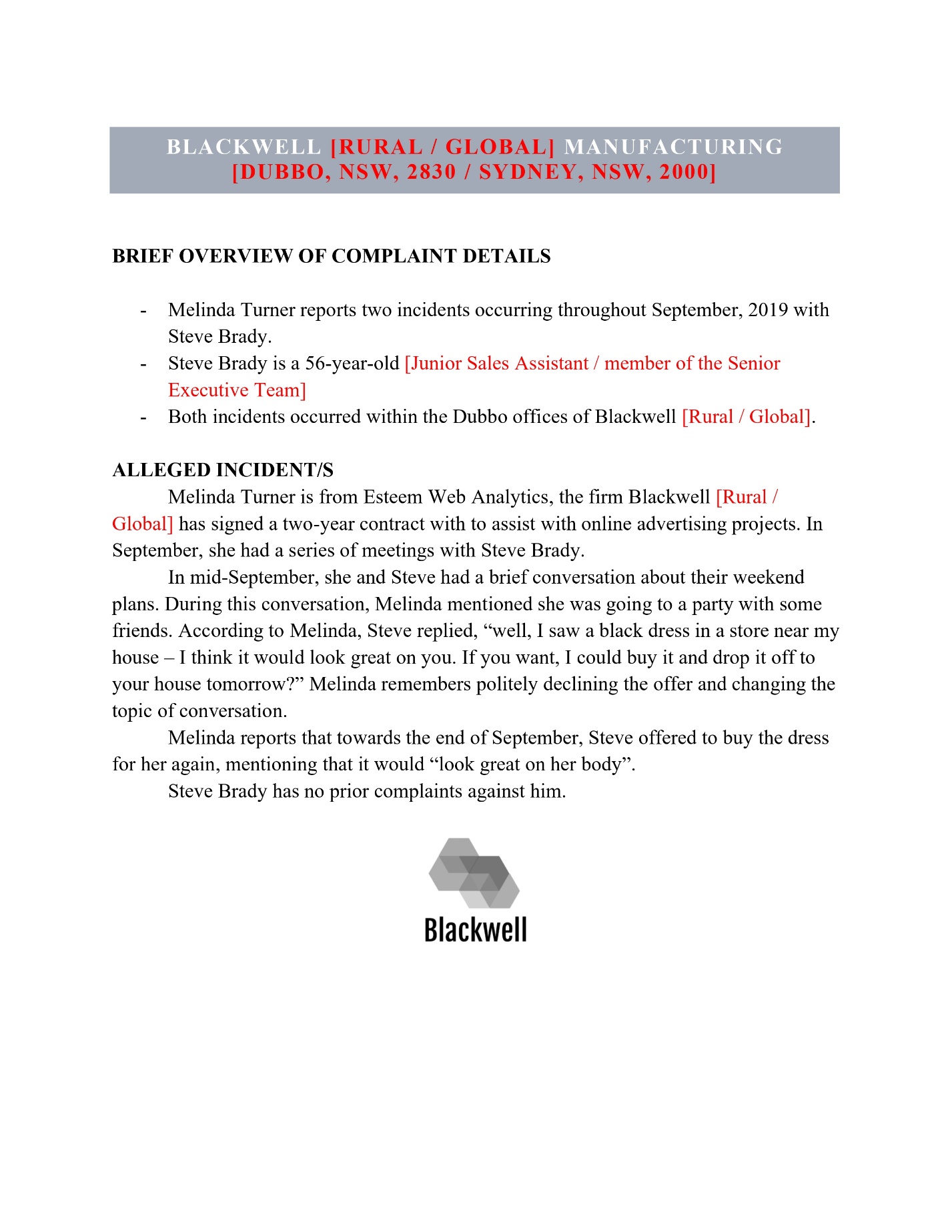
**

## 2. Vignettes used in Study 2


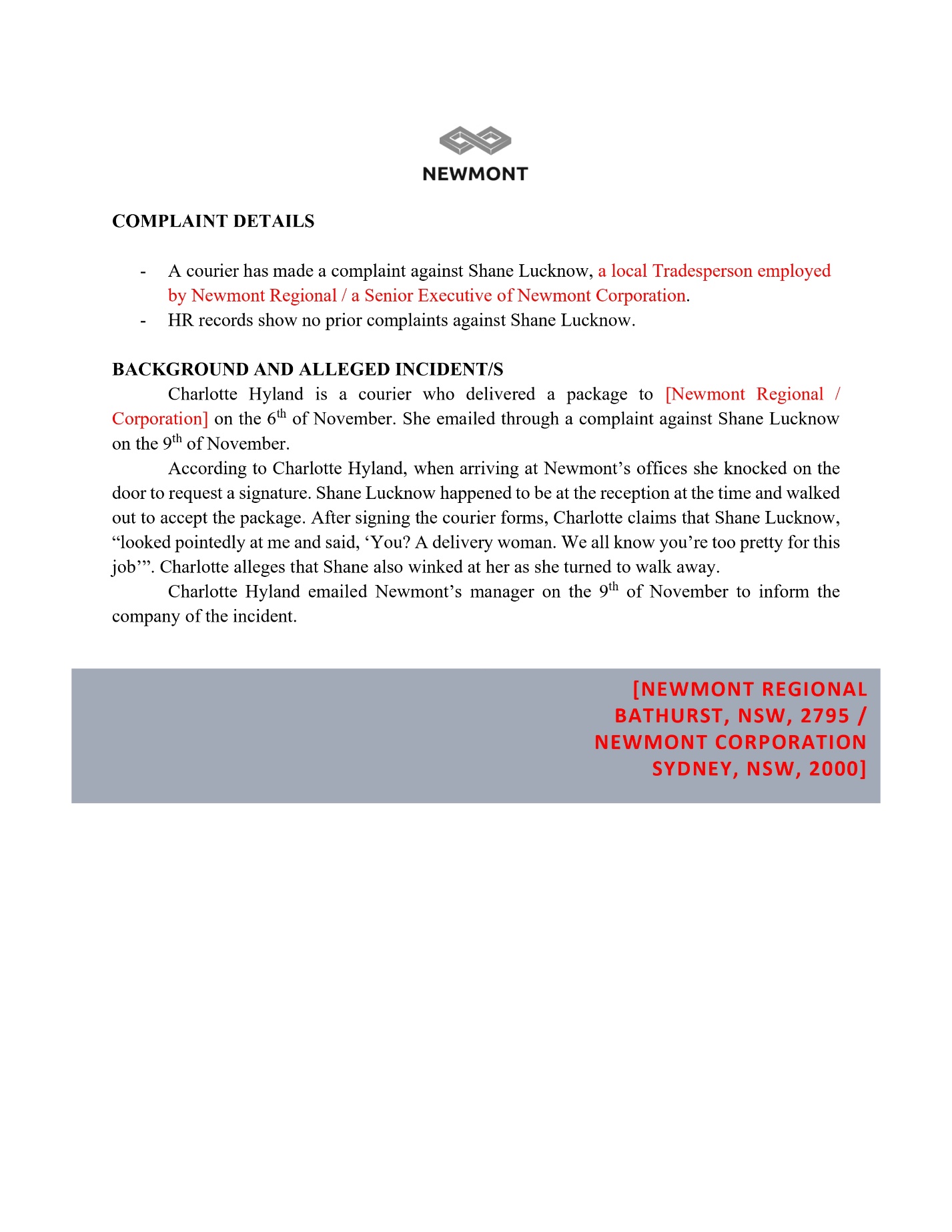


*
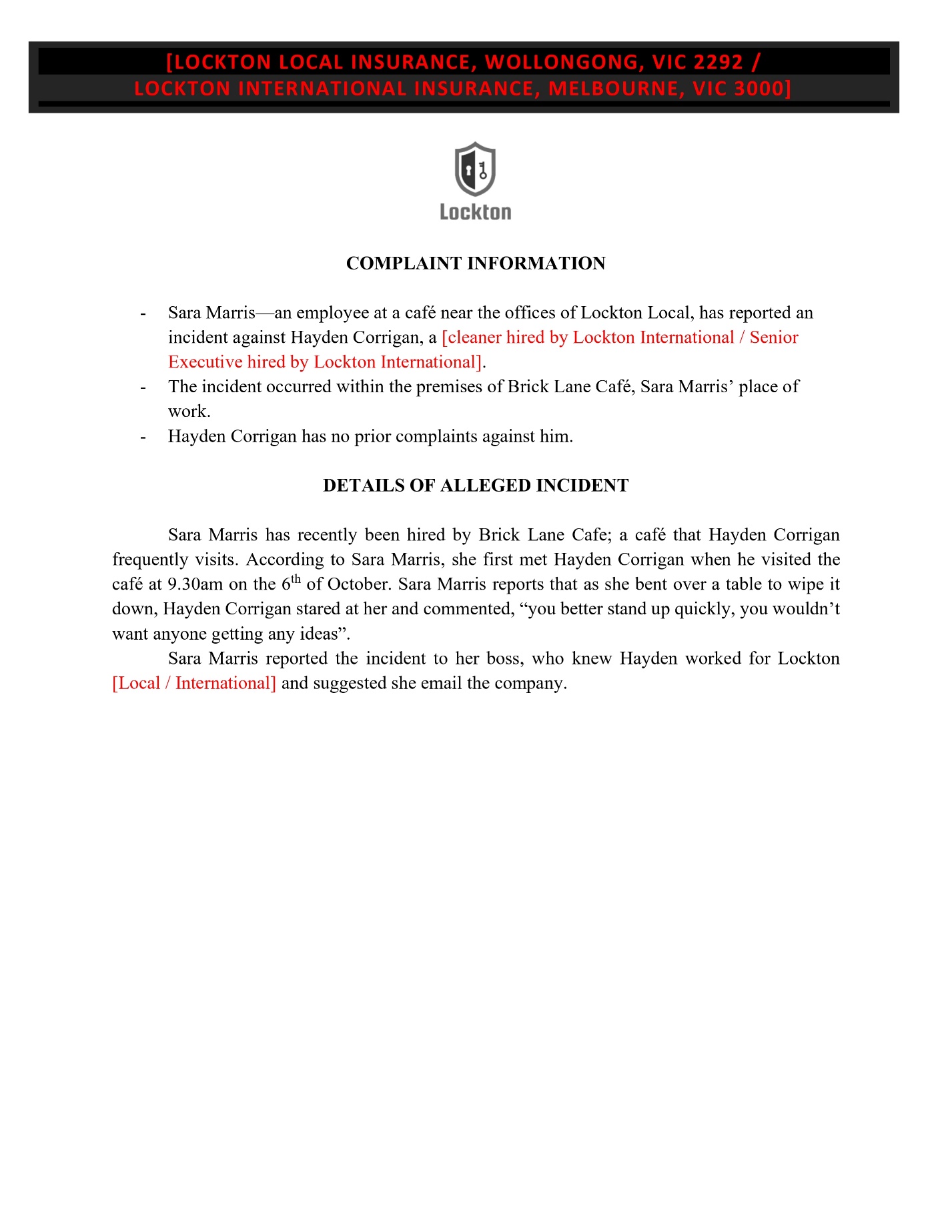
*

*
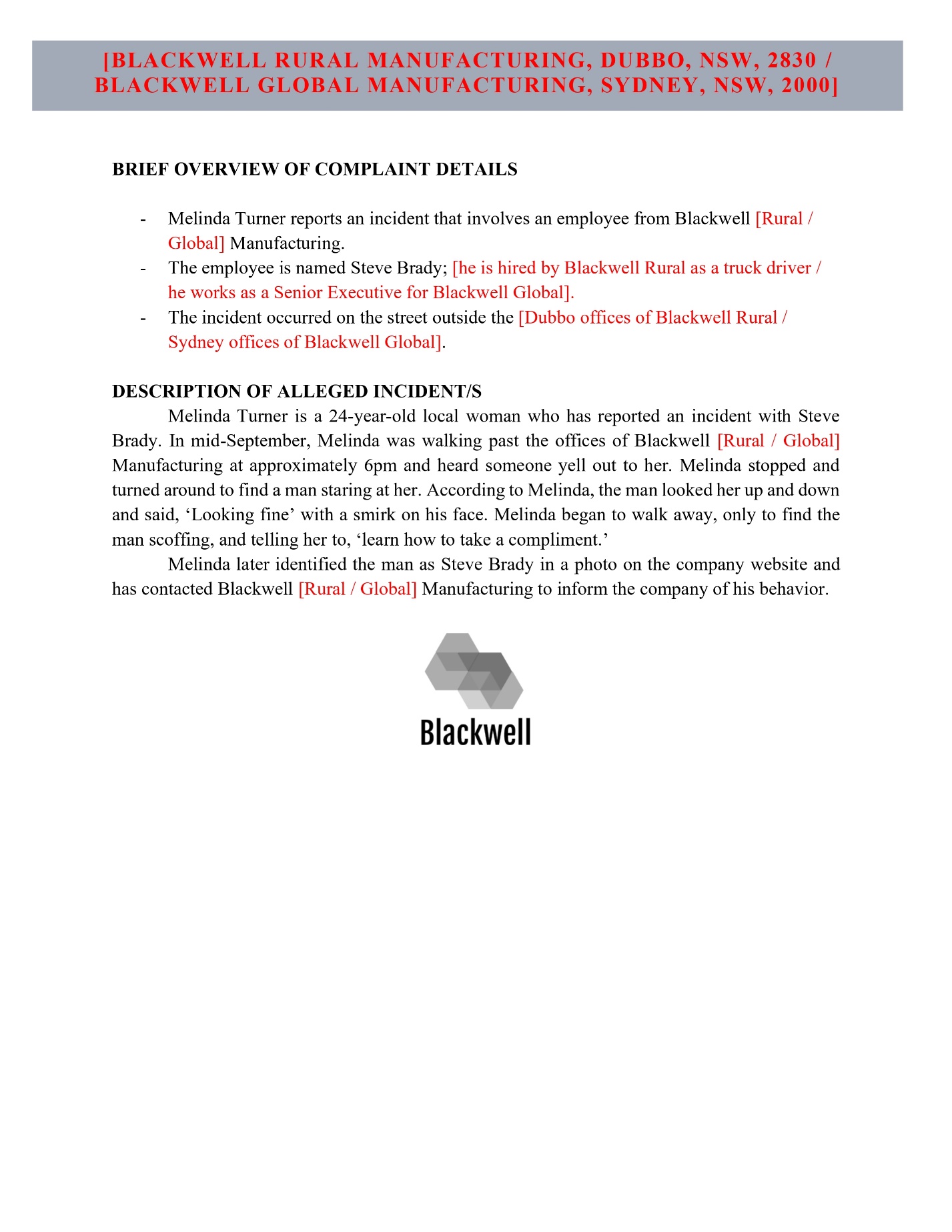
*

## 3. News articles used in Study 3


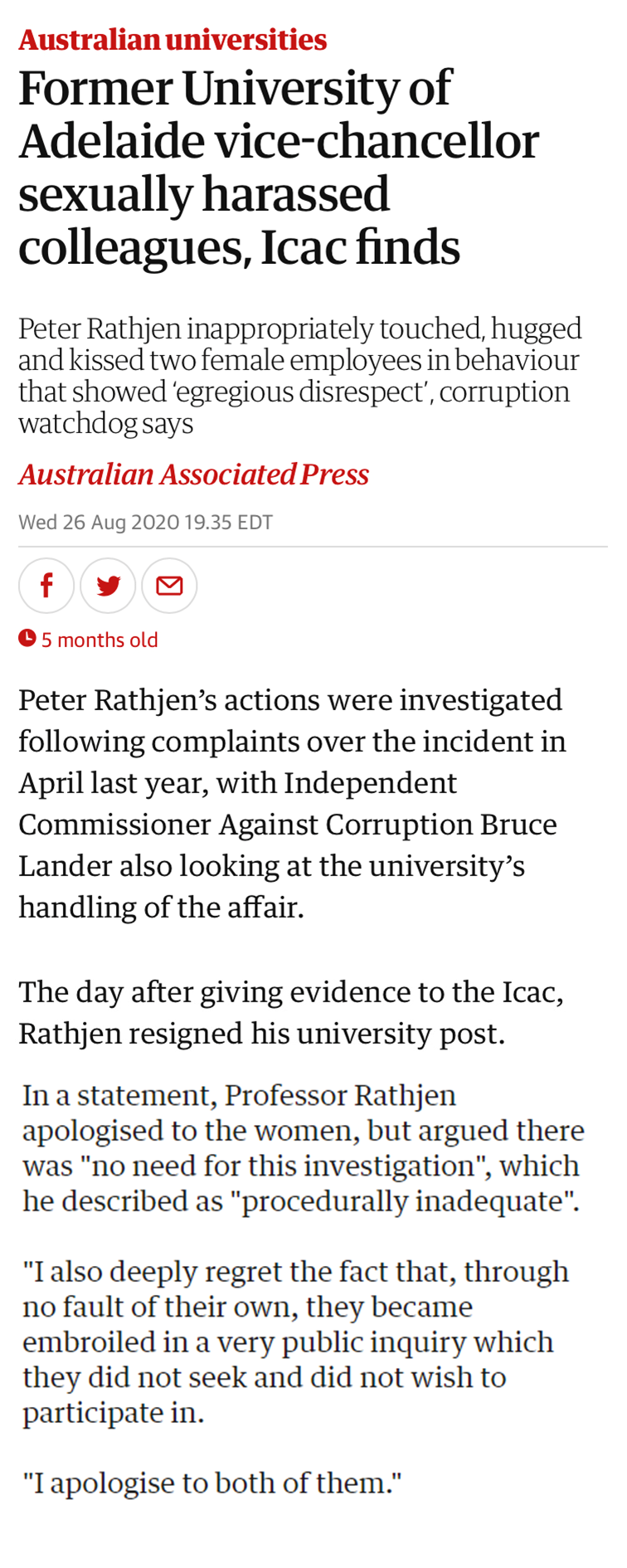


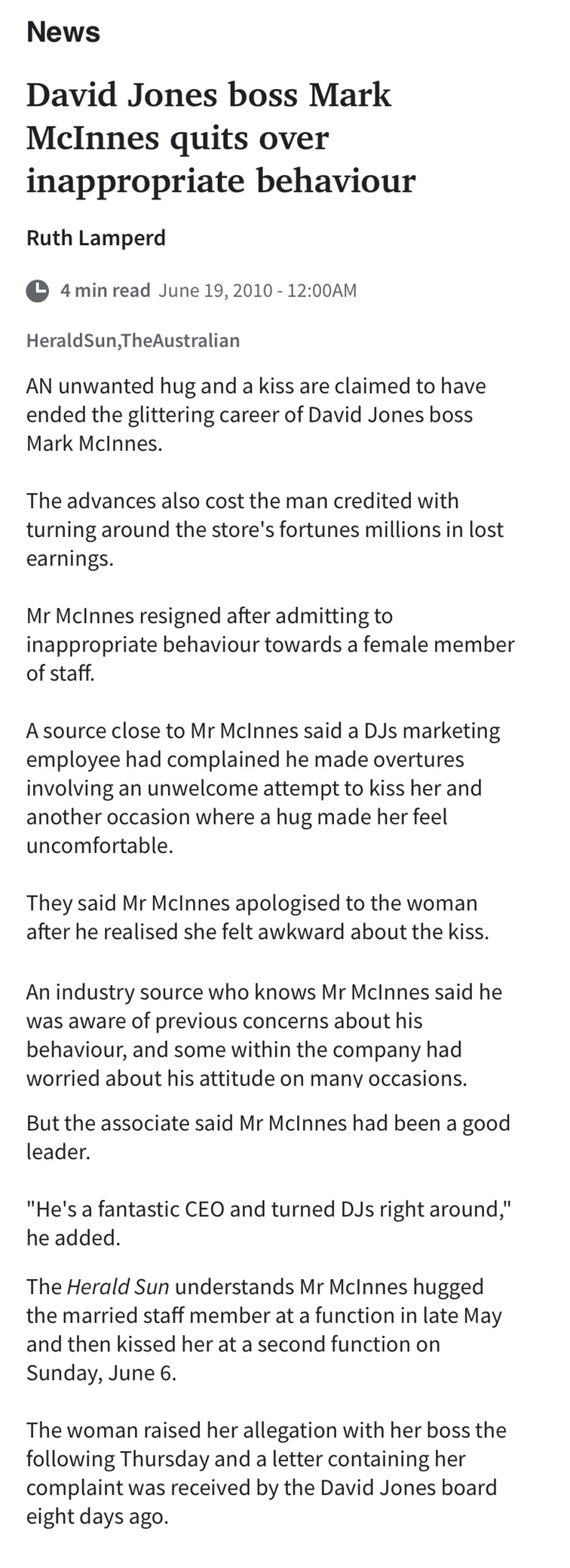


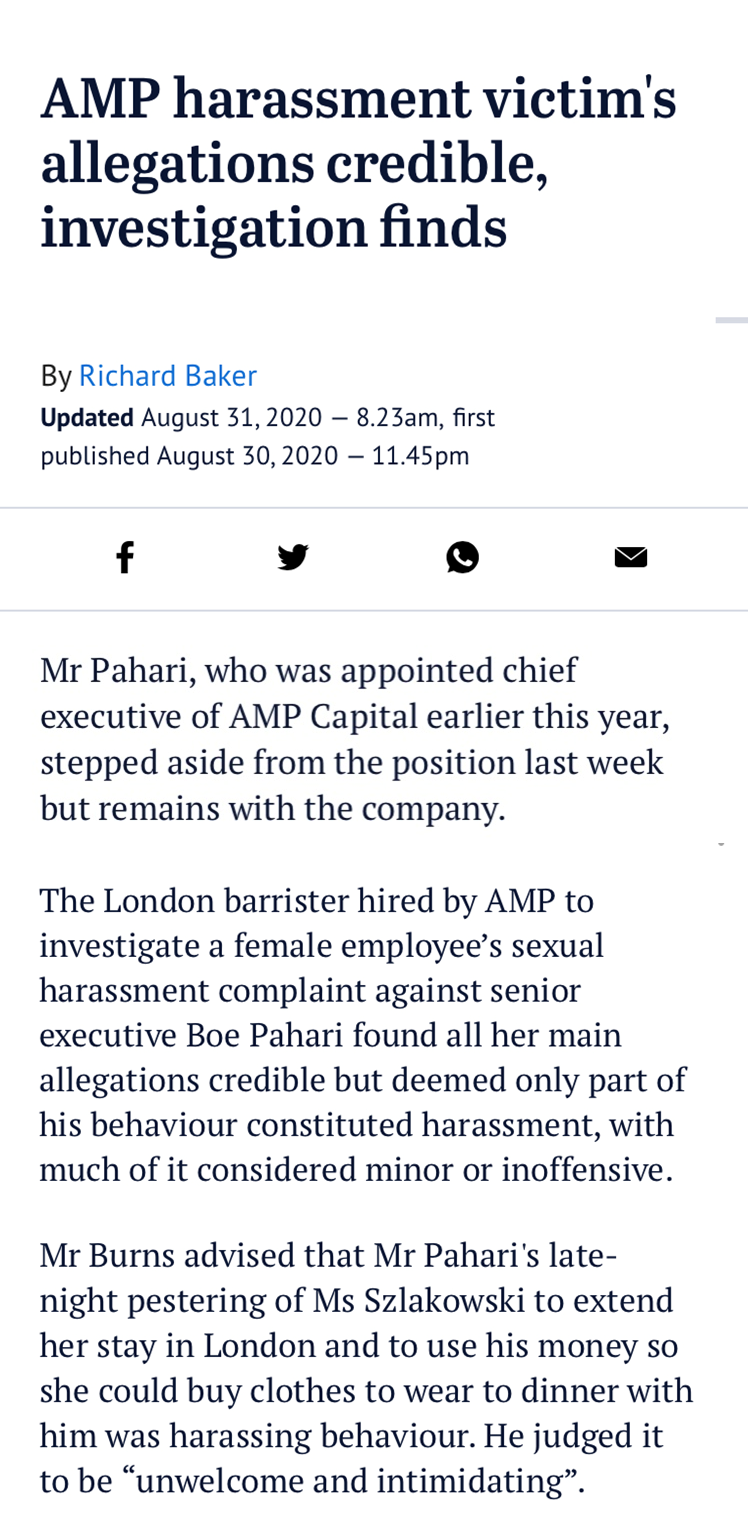


## 4. OSF and pre-registration links

OSF link: https://osf.io/hrqem/

Anonymous pre-registration for study 2: <https://aspredicted.org/blind.php?x=9BL_R2L>

Anonymous pre-registration for study 3: <https://aspredicted.org/blind.php?x=9J3_7DQ>

##

## 5. Study 1 control and outlier analyses

*Multilevel Regression Analyses with Control Variables*

**Table S1. Study 1 multilevel regression predicting perpetrator punishment from status, SDO, GSJ, and the interaction of SDO and status, including control variables.**

|  | | | | 95% CI | | |
| --- | --- | --- | --- | --- | --- | --- |
|  | ***b*** | ***SE*** | ***p*** | | **lower** | **upper** |
| Status | 0.20 | 0.13 | .146 | | -0.07 | 0.46 |
| SDO | -0.49*** | 0.06 | <.001 | | -0.61 | -0.37 |
| GSJ | -0.21*** | 0.05 | <.001 | | -0.31 | -0.11 |
| SDO x Status | -0.31** | 0.12 | .009 | | -0.54 | -0.08 |
| Control Variables |  |  |  | |  |  |
| Political Orientation | -0.01^†^ | 0.00 | .083 | | -0.01 | 0.00 |
| Gender | 0.04 | 0.14 | .777 | | -0.24 | 0.32 |
| Age | 0.00 | 0.00 | .298 | | -0.01 | 0.08 |
| Power Inequality | 0.04 | 0.02 | .105 | | -0.01 | 0.08 |
| Random effects | | |  | |  |  |
| σ2 | 1.40 |  |  | |  |  |
| τ00 ID | 0.98 |  |  | |  |  |
| τ00 vignette | 0.07 |  |  | |  |  |
| Marginal R^2^ | 0.22 |  |  | |  |  |
| Conditional R^2^ | 0.56 |  |  | |  |  |

*Note*. ^†^*p* <.10 **p* <.05, ***p* <.01, ****p* <.001. Under random effects, σ2 is the residual variance of the model. τ00 parameters are the random intercept variances for participant. CI = confidence interval. Gender is coded as 0 = male, 1 = female; political orientation is coded as 0 = very left leaning, 100 = very right leaning.

*Moderated Mediation with Control Variables*

When including control variables, bootstrapped moderated mediation analyses demonstrated that the interaction effect of perpetrator status and SDO on punishment was significantly mediated by wrongness, (*b* = -0.07, BootSE = 0.04, BootCIs (-0.15, -0.01)), but not moral agency (*b* = -0.09, BootSE = 0.07, BootCIs (-0.23, 0.03)). For those high on SDO (+1 SD), there was no significant indirect effect of status on punishment via moral wrongness (*b* = -0.08, BootSE = 0.05, BootCIs (-0.19, 0.02)). Nor was there a significant indirect effect of status on punishment via moral wrongness for those with low levels of SDO (-1SD) (*b* = 0.08, BootSE = 0.06, Bootstrapped CIs (-0.02, 0.20)).

*Multilevel Regression Analyses Including Outlier Participant*

**Table S2. Study 1 multilevel regression predicting perpetrator punishment from status, SDO, GSJ, and the interaction of SDO and status, including outlier participant.**

|  | | | | 95% CI | | |
| --- | --- | --- | --- | --- | --- | --- |
|  | ***b*** | ***SE*** | ***p*** | | **lower** | **upper** |
| Status | 0.23^†^ | 0.13 | .083 | | -0.03 | 0.49 |
| SDO | -0.53*** | 0.06 | <.001 | | -0.65 | -0.41 |
| GSJ | -0.23*** | 0.05 | <.001 | | -0.33 | -0.14 |
| SDO x Status | -0.37** | 0.12 | .002 | | -0.61 | -0.14 |
| Random effects | | |  | |  |  |
| σ2 | 1.39 |  |  | |  |  |
| τ00 ID | 1.01 |  |  | |  |  |
| τ00 vignette | 0.07 |  |  | |  |  |
| Marginal R^2^ | 0.22 |  |  | |  |  |
| Conditional R^2^ | 0.56 |  |  | |  |  |

*Note*. ^†^*p* <.10 **p* <.05, ***p* <.01, ****p* <.001. Under random effects, σ2 is the residual variance of the model. τ00 parameters are the random intercept variances for participant. CI = confidence interval.

*Moderated Mediation with Outlier Participant*

When including the outlier participant, bootstrapped moderated mediation analyses demonstrated that the interaction effect of perpetrator status and SDO on punishment was significantly mediated by wrongness, (*b* = -0.09, BootSE=0.04, Bootstrapped CIs (-0.18, -0.02)), but not moral agency (*b* = 0.12, BootSE = 0.07, Bootstrapped CIs (-0.26, 0.00)). For those high on SDO (+1 SD), there was no significant indirect effect of status on punishment via moral wrongness (*b* = -0.07, BootSE = 0.06, Bootstrapped CIs (-0.20, 0.03)). However, there was a significant indirect effect of status on punishment via moral wrongness for those with low levels of SDO (-1SD) (*b* = 0.13, BootSE = 0.07, Bootstrapped CIs (0.02, 0.28)).

## 6. Study 2 control analysis

*Multilevel Regression Analyses with Control Variables*

**Table S3. Study 2 multilevel regression predicting perpetrator punishment from status, SDO, GSJ, and the interaction of SDO and status, including control variables.**

|  | | | | 95% CI | | |
| --- | --- | --- | --- | --- | --- | --- |
|  | ***b*** | ***SE*** | ***p*** | | **lower** | **upper** |
| Status | 0.10 | 0.14 | .471 | | -0.17 | 0.37 |
| SDO | -0.48*** | 0.07 | <.001 | | -0.61 | -0.35 |
| GSJ | -0.19*** | 0.05 | <.001 | | -0.29 | -0.09 |
| SDO x Status | -0.03 | 0.12 | .789 | | -0.27 | 0.20 |
| Political Orientation | 0.00 | 0.00 | .374 | | -0.00 | 0.01 |
| Gender | -0.06 | 0.15 | .676 | | -0.36 | 0.23 |
| Age | -0.02*** | 0.00 | <.001 | | -0.03 | -0.01 |
| Power Inequality | 0.06** | 0.02 | .005 | | 0.02 | 0.11 |
| Random effects | | |  | |  |  |
| σ2 | 1.34 |  |  | |  |  |
| τ00 ID | 1.23 |  |  | |  |  |
| τ00 vignette | 0.08 |  |  | |  |  |
| Marginal R^2^ | 0.16 |  |  | |  |  |
| Conditional R^2^ | 0.58 |  |  | |  |  |

*Note*. **p* <.05, ***p* <.01, ****p* <.001. Under random effects, σ2 is the residual variance of the model. τ00 parameters are the random intercept variances for participant. CI = confidence interval. Gender is coded as 0 = male, 1 = female; political orientation is coded as 0 = very left leaning, 100 = very right leaning.

## 7. Study 3 control and outlier analyses

*Vignettes - Multilevel Regression Analyses with Control Variables*

**Table S4. Study 3 multilevel regression predicting perpetrator punishment from status, SDO, GSJ, and the interaction of SDO and status, including control variables.**

|  | | | | 95% CI | | |
| --- | --- | --- | --- | --- | --- | --- |
|  | ***b*** | ***SE*** | ***p*** | | **lower** | **upper** |
| Status | -0.02 | 0.12 | .887 | | -0.24 | 0.21 |
| SDO | -0.51*** | 0.06 | <.001 | | -0.63 | -0.39 |
| GSJ | -0.27*** | 0.04 | <.001 | | -0.36 | -0.19 |
| SDO x Status | -0.26* | 0.11 | .017 | | -0.47 | -0.05 |
| Political Orientation | 0.00 | 0.00 | .808 | | -0.01 | 0.00 |
| Gender | 0.27* | 0.13 | .036 | | 0.02 | 0.52 |
| Age | 0.00 | 0.00 | .369 | | 0.00 | 0.01 |
| Power Inequality | 0.07*** | 0.02 | <.001 | | 0.04 | 0.11 |
| Random effects | | |  | |  |  |
| σ2 | 1.47 |  |  | |  |  |
| τ00 ID | 1.14 |  |  | |  |  |
| τ00 vignette | 0.04 |  |  | |  |  |
| Marginal R^2^ | 0.20 |  |  | |  |  |
| Conditional R^2^ | 0.55 |  |  | |  |  |

*Note*. **p* <.05, ***p* <.01, ****p* <.001. Under random effects, σ2 is the residual variance of the model. τ00 parameters are the random intercept variances for participant. CI = confidence interval. Gender is coded as 0 = male, 1 = female; political orientation is coded as 0 = very left leaning, 100 = very right leaning.

*Moderated Mediation with Control Variables*

When including control variables, bootstrapped moderated mediation analyses demonstrated that the interaction effect of perpetrator status and SDO on punishment was not significantly mediated by wrongness, (*b* = -0.01, BootSE = 0.03, BootCIs (-0.07, 0.04)), nor moral agency (*b* = -0.03, BootSE = 0.05, Bootstrapped CIs (-0.15, 0.07)).

*Vignettes - Multilevel Regression Analyses Including Outliers*

**Table S5. Study 3 multilevel regression predicting perpetrator punishment from status, SDO, GSJ, and the interaction of SDO and status, including outlier participants.**

|  | | | | 95% CI | | |
| --- | --- | --- | --- | --- | --- | --- |
|  | ***b*** | ***SE*** | ***p*** | | **lower** | **upper** |
| Status | 0.14 | 0.11 | .230 | | -0.09 | 0.36 |
| SDO | -0.52*** | 0.05 | <.001 | | -0.62 | -0.41 |
| GSJ | -0.30*** | 0.04 | <.001 | | -0.38 | -0.22 |
| SDO x Status | -0.19^†^ | 0.11 | .070 | | -0.40 | 0.02 |
| Random effects | | |  | |  |  |
| σ2 | 1.45 |  |  | |  |  |
| τ00 ID | 1.32 |  |  | |  |  |
| τ00 vignette | 0.04 |  |  | |  |  |
| Marginal R^2^ | 0.19 |  |  | |  |  |
| Conditional R^2^ | 0.58 |  |  | |  |  |

*Note*. ^†^*p*<.10, **p*<.05, ***p*<.01, ****p*<.001. Under random effects, σ2 is the residual variance of the model. τ00 parameters are the random intercept variances for participant. CI = confidence interval.

*Moderated Mediation with Outlier Participants*

When including control variables, bootstrapped moderated mediation analyses demonstrated that the interaction effect of perpetrator status and SDO on punishment was not significantly mediated by wrongness, (*b* = -0.01, BootSE = 0.03, BootCIs (-0.08, 0.06)), nor moral agency (*b* = -0.04, BootSE = 0.06, BootCIs (-0.18, 0.08)).

*News Articles - Multilevel Regression Analyses with Control Variables*

**Table S6. Study 3 multilevel regression predicting news article punishment recommendations from status, SDO, GSJ, and the interaction of SDO and status, including control variables.**

|  | | | | 95% CI | | |
| --- | --- | --- | --- | --- | --- | --- |
|  | ***b*** | ***SE*** | ***p*** | | **lower** | **upper** |
| SDO | -0.46*** | 0.08 | <.001 | | -0.61 | -0.31 |
| GSJ | -0.16** | 0.05 | .002 | | -0.27 | -0.06 |
| Political Orientation | 0.00 | 0.00 | .123 | | -0.00 | -0.01 |
| Gender | 0.24 | 0.16 | .124 | | -0.07 | 0.55 |
| Age | 0.01** | 0.00 | .009 | | 0.00 | 0.02 |
| Case Familiarity | 0.05** | 0.02 | .016 | | 0.01 | 0.09 |
| Random effects | | |  | |  |  |
| σ2 | 1.76 |  |  | |  |  |
| τ00 ID | 1.91 |  |  | |  |  |
| τ00 vignette | 0.09 |  |  | |  |  |
| Marginal R^2^ | 0.08 |  |  | |  |  |
| Conditional R^2^ | 0.57 |  |  | |  |  |

*Note*. **p* <.05, ***p* <.01, ****p* <.001. Under random effects, σ2 is the residual variance of the model. τ00 parameters are the random intercept variances for participant. CI = confidence interval. Gender is coded as 0 = male, 1 = female; political orientation is coded as 0 = very left leaning, 100 = very right leaning.

*News Articles - Multilevel Regression Analyses Including Outliers*

**Table S7. Study 3 multilevel regression predicting news article punishment recommendations from status, SDO, GSJ, and the interaction of SDO and status, including outlier participants.**

|  | | | | 95% CI | | |
| --- | --- | --- | --- | --- | --- | --- |
|  | ***b*** | ***SE*** | ***p*** | | **lower** | **upper** |
| SDO | -0.51*** | 0.07 | <.001 | | -0.64 | -0.37 |
| GSJ | -0.15*** | 0.05 | .004 | | -0.25 | -0.05 |
| Random effects | | |  | |  |  |
| σ2 | 1.73 |  |  | |  |  |
| τ00 ID | 2.12 |  |  | |  |  |
| τ00 vignette | 0.09 |  |  | |  |  |
| Marginal R^2^ | 0.09 |  |  | |  |  |
| Conditional R^2^ | 0.60 |  |  | |  |  |

*Note*. **p* <.05, ***p* <.01, ****p* <.001. Under random effects, σ2 is the residual variance of the model. τ00 parameters are the random intercept variances for participant. CI = confidence interval.

## 8. Integrative data analysis with control variables

**Table S8. Multilevel Bayesian linear model investigating status, SDO, GSJ and the interaction of SDO and status as predictors of punishment recommendations for sexual harassment vignettes across three studies, including control variables.**

|  | | | 95% CI | |
| --- | --- | --- | --- | --- |
|  | ***b*** | ***SE*** | **lower** | **upper** |
| Status | 0.10 | 0.07 | -0.04 | 0.25 |
| SDO | -0.50 | 0.04 | -0.57 | -0.42 |
| GSJ | -0.24 | 0.03 | -0.29 | -0.18 |
| SDO x Status | -0.19 | 0.07 | -0.33 | -0.07 |
| Political Orientation | 0.00 | 0.00 | -0.01 | 0.01 |
| Gender | 0.09 | 0.08 | -0.07 | 0.25 |
| Age | -0.01 | 0.00 | -0.02 | 0.01 |
| Power Inequality | 0.06 | 0.01 | 0.03 | 0.08 |
| Random effects | | |  |  |
| σ2 | 1.41 |  |  |  |
| τ00 ID | 1.13 |  |  |  |
| τ00 study | <0.01 |  |  |  |
| τ00 vignette | 0.13 |  |  |  |
| τ11 study.SDO | <0.01 |  |  |  |
| τ11 study.Status | <0.01 |  |  |  |
| τ11 study.GSJ | <0.01 |  |  |  |
| τ11 study.SDO x Status | <0.01 |  |  |  |
| τ11 study.Political Orientation | <0.01 |  |  |  |
| τ11 study.Gender | <0.01 |  |  |  |
| τ11 study.Age | <0.01 |  |  |  |
| τ11 study.Power Inequality | <0.01 |  |  |  |
| Marginal R^2^ | 0.16 |  |  |  |
| Conditional R^2^ | 0.62 |  |  |  |

*Note*. Under random effects, σ2 is the residual variance of the model. τ00 parameters are the random intercept variances for participant, study, and vignette, τ11 are the random slope variances for the predictors. CI = confidence interval. Gender is coded as 0 = male, 1 = female; political orientation is coded as 0 = very left leaning, 100 = very right leaning.

## 9. Integrative data analysis with outlier participants

**Table S9. Multilevel Bayesian linear model investigating status, SDO, GSJ and the interaction of SDO and status as predictors of punishment recommendations for sexual harassment vignettes across three studies, including outlier participants.**

|  | | | 95% CI | |
| --- | --- | --- | --- | --- |
|  | ***b*** | ***SE*** | **lower** | **upper** |
| Status | 0.19 | 0.08 | 0.04 | 0.35 |
| SDO | -0.49 | 0.04 | -0.57 | -0.41 |
| GSJ | -0.26 | 0.03 | -0.33 | -0.19 |
| SDO x Status | -0.20 | 0.07 | -0.34 | -0.06 |
| Random effects | | |  |  |
| σ2 | 1.41 |  |  |  |
| τ00 ID | 1.24 |  |  |  |
| τ00 study | <0.001 |  |  |  |
| τ00 vignette | 0.19 |  |  |  |
| τ11 study.SDO | <0.01 |  |  |  |
| τ11 study.status | <0.01 |  |  |  |
| τ11 study.GSJ | <0.01 |  |  |  |
| τ11 study.SDO x status | <0.01 |  |  |  |
| Marginal R^2^ | 0.17 |  |  |  |
| Conditional R^2^ | 0.60 |  |  |  |

*Note*. Under random effects, σ2 is the residual variance of the model. τ00 parameters are the random intercept variances for participant, study, and vignette, τ11 are the random slope variances for the predictors. CI = confidence interval. Gender is coded as 0 = male, 1 = female; political orientation is coded as 0 = very left leaning, 100 = very right leaning.
